# Calorimetric study of water's two glass transitions in the presence of LiCl

**DOI:** 10.1039/c7cp08677f

**Published:** 2018-02-07

**Authors:** Guadalupe N. Ruiz, Katrin Amann-Winkel, Livia E. Bove, Horacio R. Corti, Thomas Loerting

**Affiliations:** a Institute of Physical Chemistry , University of Innsbruck , Innrain 52c , 6020 Innsbruck , Austria . Email: thomas.loerting@uibk.ac.at; b Departament de Física e Enginyeria Nuclear , Universitat Politècnica de Catalunya , 08028 , Barcelona , Spain; c Department of Physics , AlbaNova University Center , 10691 Stockolm , Sweden; d Institut de Mineralogie et de Physique des Milieux Condenses , CNRS-Universitè P.et M. Curie , 4 place de Jussieu , 75005 Paris , France; e Institute of Condensed Matter Physics , Ecole Polytechnique Fédérale de Lausanne , Lausanne , Switzerland; f Departamento de Física de la Materia Condensada , Comisión Nacional de Energía Atómica , San Martín , Buenos Aires , Argentina; g Instituto de Química Física de los Materiales , Medio Ambiente y Energía , Universidad de Buenos Aires , Ciudad Autónoma de Buenos Aires , Argentina

## Abstract

A DSC study of dilute glassy LiCl aqueous solutions in the water-dominated regime provides direct evidence of a glass-to-liquid transition in expanded high density amorphous (eHDA)-type solutions. Similarly, low density amorphous ice (LDA) exhibits a glass transition prior to crystallization to ice I_c_. Both glass transition temperatures are independent of the salt concentration, whereas the magnitude of the heat capacity increase differs. By contrast to pure water, the glass transition endpoint for LDA can be accessed in LiCl aqueous solutions above 0.01 mole fraction. Furthermore, we also reveal the endpoint for HDA's glass transition, solving the question on the width of both glass transitions. This suggests that both equilibrated HDL and LDL can be accessed in dilute LiCl solutions, supporting the liquid–liquid transition scenario to understand water's anomalies.

## Abstract 2

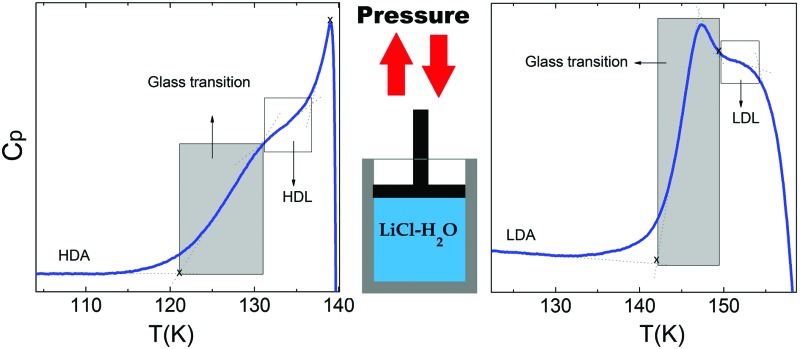
Based on calorimetric data we show that water's two distinct glass transitions can be accessed up to the endpoint in dilute LiCl solutions. By contrast, in pure water both endpoints are masked.

## 1 Introduction

**Fig. 1:**
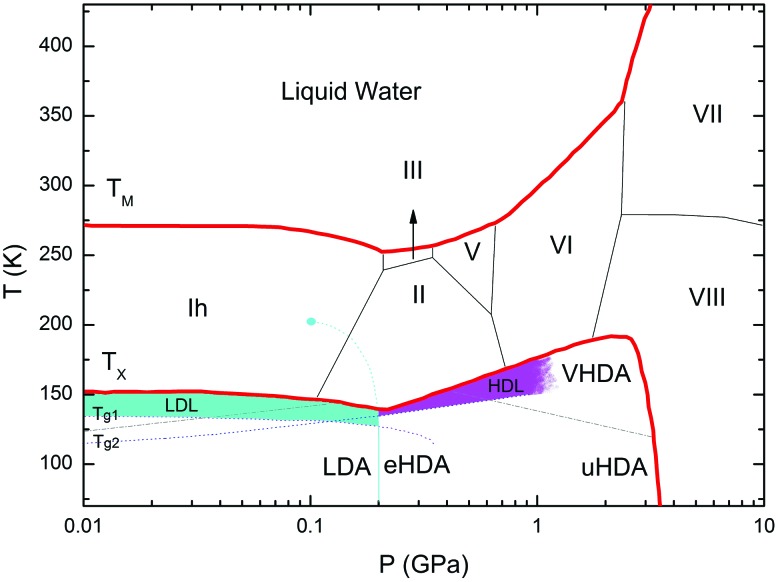
Phase diagram of non-crystalline water including the (metastable) amorphous ices LDA, HDA and VHDA, surrounded by the thick red crystallization line *T*_*x*_.^21^*T*_M_ stands for melting temperature and roman numbers stand for the crystalline phases of ice. The light-blue solid line separating LDA and HDA was taken from Fig. 3 in ref. 15, whereas the grey dash-dotted line between HDA and VHDA was deduced from Fig. 3(b) in ref. 10. Two ultraviscous liquid domains, low- and high-density liquid water (LDL and HDL), can be found just below *T*_*x*_. The two corresponding glass transition temperatures *T*_g1_ and *T*_g2_ separating the glassy solids LDA and HDA from the ultraviscous liquids LDL and HDL are taken from ref. 22 and 23, respectively. Note the metastable extension of *T*_g1_ into the stability region of HDA and of *T*_g2_ into the stability region of LDA/LDL (figure adapted from ref. 19 and 20).

Water, the most ubiquitous and essential compound on Earth, exhibits many anomalies that make it unique as compared to other simple substances. Water's polymorphism, the existence of a large number of crystalline ices over a wide range of temperature and pressure, is one of the peculiar properties of water. Although amorphous ice is the most abundant form of water in the interstellar space,^1^ the polyamorphism of water is a relatively novel and intriguing subject in the physics of condensed matter. Water displays three different amorphous ices, namely low-(LDA), high-(HDA) and very high-density amorphous ice (VHDA),^2^,^3^ which can be prepared in the laboratory.^4^ These amorphous ices can be inter-converted under pressure, where jump-like transitions set the physics apart from traditional glass physics. Annealing procedures lead to more relaxed glassy states that exhibit higher thermal stability.^5^–^7^ In the case of HDA, several distinct preparation procedures have been reported: Unannealed HDA (uHDA) was firstly prepared by Mishima *et al.*^8^ by pressure induced amorphization of hexagonal ice at 77 K and pressures above 1 GPa. It represents the most experimentally studied form of HDA until recently. Expanded HDA (eHDA), on the other hand, is an annealed form of HDA, which has been the object of by far fewer investigations.^6^,^7^,^9^,^10^ Although uHDA does not show a calorimetric glass transition, eHDA presents one at 116 K.^11^ Whether or not this glass transition involves the liquid-like translational mobility of water molecules is still a matter of debate.^11^–^14^ It has been argued that HDA and LDA could be solid proxies of distinct liquid states in the deeply supercooled regime, namely HDL (high density liquid) and LDL (low density liquid), respectively.^15^ Different interpretations about the same phenomenology not only prevail in experiments, but also in simulation work on HDA.^16^–^18^ Poole *et al.* have proposed, through a classical molecular dynamics simulation using the ST2 model,^17^ that a line of first-order transition between these two liquids exists and ends at a second (liquid–liquid) critical point (LLCP), below the line of homogeneous ice nucleation. Thus, this coexistence line, located deep in the water supercooled region (no man's land), is inaccessible. Fig. 1, derived from phase diagrams presented in ref. 19 and 20, includes the metastable amorphous phases LDA, HDA and VHDA, the critical point (LLCP) and the proposed LDL and HDL regions as well as the *T*_g_ lines connecting them to LDA and HDA, respectively.

One key open question regarding both glass transitions is the question about their endpoints, *i.e.*, the temperatures above which the liquids can be regarded as equilibrated as opposed to approaching equilibrium. This is because the glass transitions observed in the literature are terminated prior to reaching the endpoint, *e.g.*, terminated by crystallization in the case of LDL. For this reason, the question of the true width of water's glass transition(s) has been discussed vividly^24^–^27^ for decades, but still not solved.

One way of approaching this question is by using aqueous solutions rather than pure water. This has not been carried out at all for eHDA solutions, and is hence the topic of the present work. Angell and coworkers pioneered the study of liquid–liquid immiscibility and polyamorphism using LiCl aqueous solutions, which easily vitrify upon cooling, avoiding crystallization.^28^–^30^ Yoshimura and Kanno performed detailed Raman spectroscopy studies on LiCl aqueous solutions and suggested the existence of a transition from the relaxed amorphous phase to the supercooled liquid at high pressures and low temperatures.^31^ These studies were impeded by the non-glass forming tendency of dilute aqueous solutions through vitrification of the liquid. This obstacle is overcome here by resorting to pressure-induced amorphization as the process used for formation of glassy solutions. The detailed studies of LiCl aqueous solutions as a function of composition and pressure were performed by Mishima and Suzuki with the aim of finding the link between LDA–HDA polyamorphism and liquid–liquid immiscibility.^32^–^37^ More recently, Suzuki and Mishima^38^,^39^ extended the pressure-induced amorphization studies to glycerol aqueous solutions in order to obtain further evidence of the existence of the LLCP in solvent water. Mishima conjectured that the solvent water in aqueous solutions is structurally related to HDL, rather than to LDL.^34^ This hypothesis was later endorsed by other studies,^40^–^42^ and a well vitrified system, structurally similar to pure eHDA was obtained by cooling the eutectic solution at standard cooling rates. The observation of the HDL–LDL transition in calorimetric studies of ionic liquids has recently been reported by Zhao and Angell.^43^

In a previous study on pressure-induced amorphization and polyamorphism in LiCl aqueous solutions,^44^ we showed that uHDA is formed by compression to 1.6 GPa at 77 K in the sub-eutectic concentration range (salt mole fraction, *x* < 0.125), or water-dominated regime, as a result of the amorphization of segregated water.^45^ In contrast, the salt-dominated regime (*x* > 0.125) exhibits a broad densification of the sample due to the segregation of patches of LiCl hydrates within the glassy LiCl matrix. In our earlier study, we focused our attention on the unannealed state of HDA (uHDA), which does not show a glass transition at 1 bar, either in pure water or salty samples. We here focus our attention on the pressure-annealed, expanded HDA (eHDA) that shows a glass transition for pure water. In a narrow interval around the eutectic composition, a well vitrified system, structurally similar to pure eHDA, was obtained by cooling the solution at standard cooling rates.^40^

Differential Scanning Calorimetry (DSC) heating scans of recovered samples after pressurization (Fig. 9 in ref. 44) indicate that the onset of the polyamorphic HDA → LDA transition, at 121 K, is not affected by the salt content up to *x* = 0.03, whereas the transition becomes increasingly broader up to *x* = 0.12, and is absent for *x* > 0.14. The heat released at the transition indicates that the hydration water also experiences a HDA → LDA transition. In addition to signatures of the polyamorphic transition, the calorimetry scans also reveal signatures of glass–liquid transitions: the glass–liquid transition of the unfreezable (eutectic) LiCl solution was clearly observed at 140 K for samples with *x* = 0.13–0.14, while very weak signatures of this transition were also seen for *x* = 0.05–0.11. Just like in pure uHDA, no glass transition related to the transformation of the amorphous solid (HDA) to the ultraviscous, supercooled liquid (HDL) could be detected in samples of *x* < 0.05. The reason for the absence of the glass transition is that it is masked by the exotherm at the polyamorphic HDA → LDA transition. In order to disentangle the two effects, our strategy was to relax the HDA sample, thereby shifting the HDA → LDA transition at 1 bar to higher temperature, allowing for a direct observation of the glass-to-liquid transition of the HDA patches in the salty sample, which is the same strategy used also in ref. 11 for pure water. This requires a more complex sample preparation leading to the formation of eHDA, including high-pressure annealing and high-temperature (140 K) decompression to relax the sample. In samples prepared in this way, we see evidence of the existence of both HDL and LDL, and their link to the amorphous phases through two distinct glass transitions.

## 2 Experimental section

eHDA samples were prepared by pressure induced amorphization using a material testing machine (Zwick, model BZ100/TL3S) as already described.^6^ The machine applies a vertical force (max. 100 kN) at a controlled rate, and the position of the piston is recorded with a reproducibility of ±0.5 μm and a spatial resolution of 0.01 μm. The liquid sample (500 μl) is pipetted into an indium container and placed inside a cylindrical stainless steel cell that is pressed by a combination of stainless steel pistons. Temperature is regulated with heaters inside the cell, and copper loops located around it, which allow the flow of liquid nitrogen. A Pt100 sensor is located inside the cell to control the temperature.

**Fig. 2:**
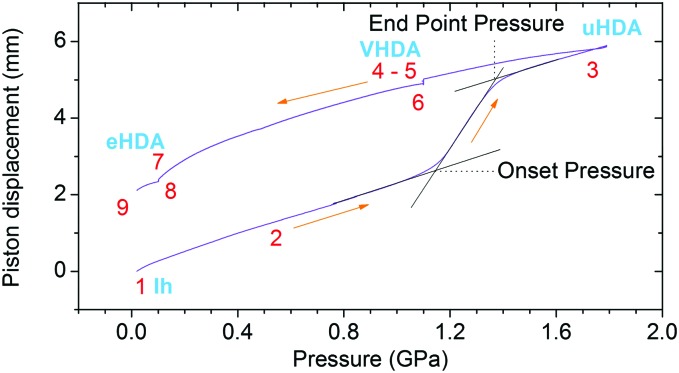
Piston displacement as a function of pressure for the steps in Table 1.

Table 1 shows the pressure–temperature steps followed to prepare eHDA, while Fig. 2 illustrates the corresponding piston displacement as a function of pressure. The piston displacement represents the change in sample thickness (volume), *i.e.*, it is a measure of density change.

**Table 1:** Temperature–pressure steps for the preparation of eHDA samples. *S* indicates the step number, *T*_i_, *T*_f_, *p*_i_ and *p*_f_ stand for initial and final temperature and pressure respectively, whereas 
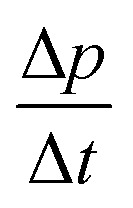
 is the compression/decompression rate

S	*T*_i_ → *T*_f_ (K)	*p*_i_ → *p*_f_ (GPa)	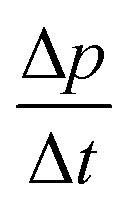 (MPa min^–1^)	Process
1	300 → 77	0	—	Isobaric cooling
2	77	0 → 0.7	140	Isotherm. comp.
3	77	0.7 → 1.8	20	uHDA formation
4	77	1.8 → 1.1	140	Isotherm. decomp.
5	77 → 160	1.1	—	VHDA formation
6	160 → 140	1.1	—	Isobaric cooling
7	140	1.1 → 0.1	20	eHDA formation
8	140 → 77	0.1	—	Quenching
9	77	0.1 → 0	140	Pressure release

LDA samples were prepared by isobaric heating of eHDA inside a Differential Scanning Calorimeter (DSC). All XRD measurements were carried out with a commercial powder X-ray diffractometer (Siemens, model D5000) equipped with a low-temperature Anton Paar chamber. The sample holder made of nickel-plated copper can be cooled to ∼80 K with liquid nitrogen and controlled up to room temperature. The diffractograms were recorded using an incident wavelength of *λ* = 1.54178 Å (CuKα). Samples were powdered under liquid nitrogen and quickly transferred onto the pre-cooled sample holder to minimize water vapor condensation.

DSC scans were recorded using a PerkinElmer DSC 8000. The samples were loaded inside aluminum capsules, which were manually closed with a lid of the same material. This process was carried out under liquid nitrogen so that weighing the samples was not possible and their mass had to be calculated from each corresponding melting exotherm as explained in our previous publication.^44^ Two different protocols were used for these studies: with and without annealing of the eHDA sample. In the latter case, samples were scanned at 30 K min^–1^ from 93 to 253 K, recooled and scanned again from 93 to 313 K to melt the sample. In the former case, the HDA-type samples were annealed for 90 minutes at 108 K (a few K below *T*_g_) and then the glass transition of HDA was scanned twice from 93 to 123 K. HDA was then converted to LDA by heating to 145 K and keeping the temperature constant for 10 minutes, after which the glass transition of LDA was scanned by heating from 93 K – all at rates of 30 K min^–1^.

## 3 Results

### 3.1 Dilatometric study

The piston displacement curves like the one shown in Fig. 2 were found to be highly similar to the pure water case reported in our earlier work up to mole fractions of 0.103. Thus, the addition of LiCl does not significantly affect the phase behaviour, *i.e.*, pressure-induced amorphization and polyamorphic transitions. Thus, the nomenclature employed for pure water is also appropriate for LiCl solutions up to mole fractions of 0.103.

### 3.2 XRD characterization

**Fig. 3:**
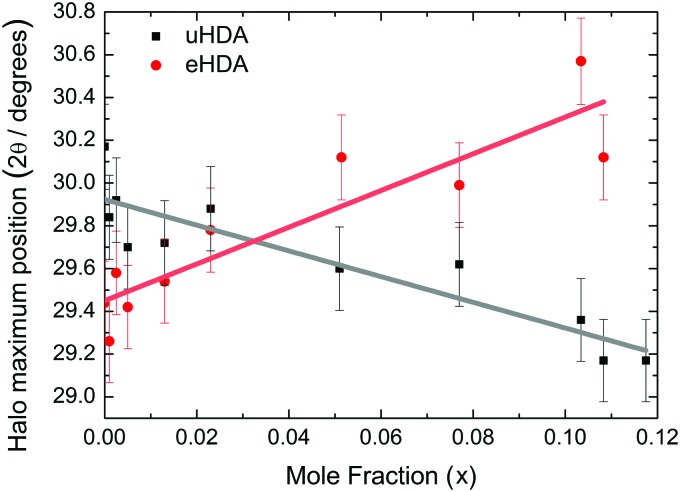
Position of halo maximum as a function of the LiCl mole fraction obtained from XRD of eHDA samples (red dots) and uHDA samples (black squares), reported in ref. 44. Error bars are derived from the uncertainty when placing the position of the halo maximum and reproducibility of the results.

Fig. 3 shows the position of the halo maximum as a function of LiCl concentration for all studied eHDA samples and for all uHDA samples previously studied and reported in ref. 44. Straight lines correspond to the linear fits. It is well known that for pure water samples, the position of the halo maximum correlates with density, see for example Fig. 5 in ref. 46. In addition, the halo position also shifts because of the amount of LiCl contained in the amorphous matrix. However, for same amounts of LiCl, there is a slight difference in the position of the halo maximum. For pure water, the uHDA halo appears at angles (2*θ*) higher by 0.5 ± 0.3° than for eHDA, reflecting the slightly expanded nature of eHDA. For a mole fraction of *x* = 0.10, however, the uHDA halo appears lower by 1.0 ± 0.3° than for eHDA. Even though the effect itself is small, close to the resolution limit, it still testifies that relaxation effects towards a more stable glassy state take place within the sample upon decompression at 140 K. These relaxation effects are further investigated using DSC.

### 3.3 Calorimetric study

#### 3.3.1 Water's second glass transition: HDA → HDL

**Fig. 4:**
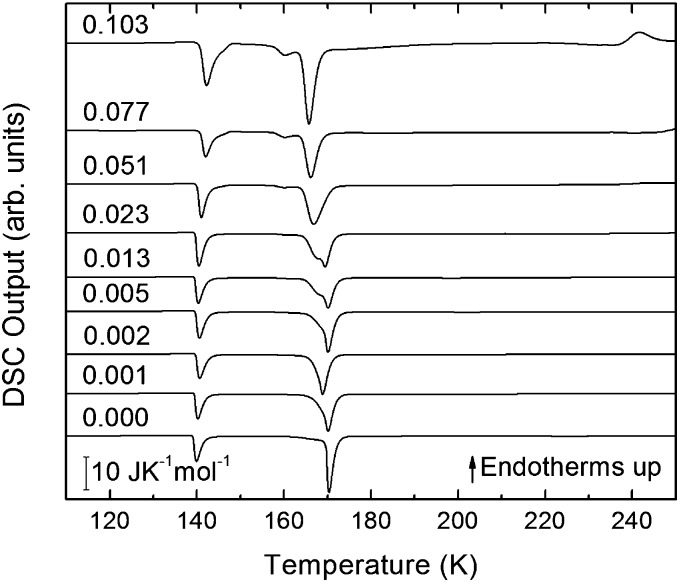
DSC scans (heating rate 30 K min^–1^) of recovered samples after pressurization for mole fractions from *x* = 0 to *x* = 0.103.

The single DSC scans for eHDA samples without annealing inside the DSC instrument at 1 bar are shown in Fig. 4 in the water-dominated concentration regime.

The exothermic eHDA–LDA transition takes place at the onset temperature of 135 K, that is, approximately 15 K above that observed for the uHDA → LDA transition,^44^ due to the fact that eHDA is a more relaxed and stable phase. The second exotherm, corresponding to the transition from LDA to cubic ice can be observed around 170 K for pure water, and shifts down to 160 K with increasing salt mole fraction.

**Fig. 5:**
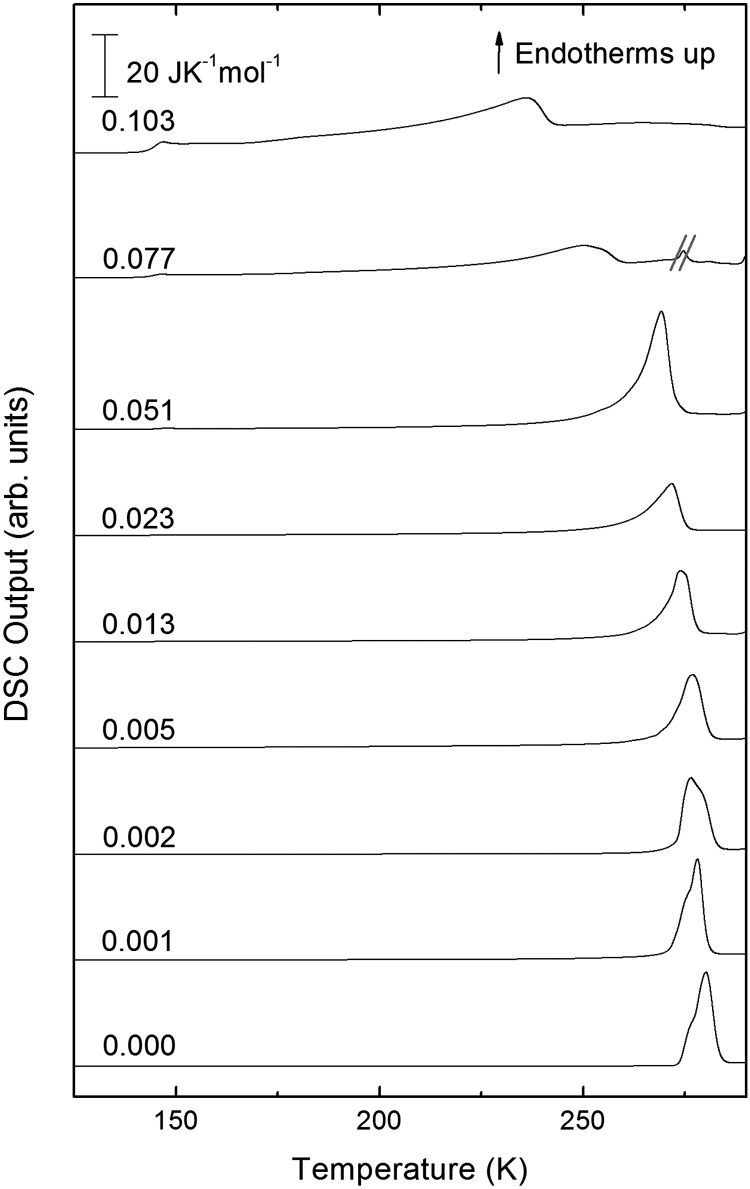
DSC scans of melting endotherms taken at 30 K min^–1^ of samples shown in Fig. 4 for mole fractions from *x* = 0 to *x* = 0.103. The additional peak in sample *x* = 0.077 has been crossed out because it is an artifact of the measurement.

**Fig. 6:**
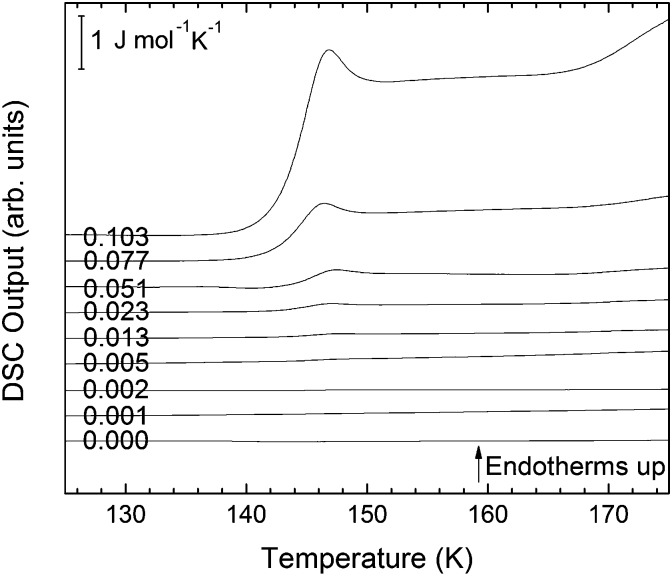
Magnification of Fig. 5 in the temperature range between 125 and 175 K for mole fractions from *x* = 0 to *x* = 0.103.

The second scan including the melting is shown in Fig. 5. As expected from the phase diagram shown in Fig. 1 of ref. 44, the melting endotherms of the samples shift to lower temperatures with increasing LiCl concentrations, and no additional peak is observed in any case, indicating the absence of phase segregation. The zoom in between 125 and 175 K (Fig. 6), however, shows the presence of eutectic patches of LiCl–H_2_O for *x* > 0.05, which show a glass transition at about 142 K (*T*_g_ onset) even after melting and recooling of the sample. The step in heat capacity amounts to about 0.5 J K^–1^ mol^–1^ for the solution of 0.051 and increases with mole fraction of LiCl.

**Fig. 7:**
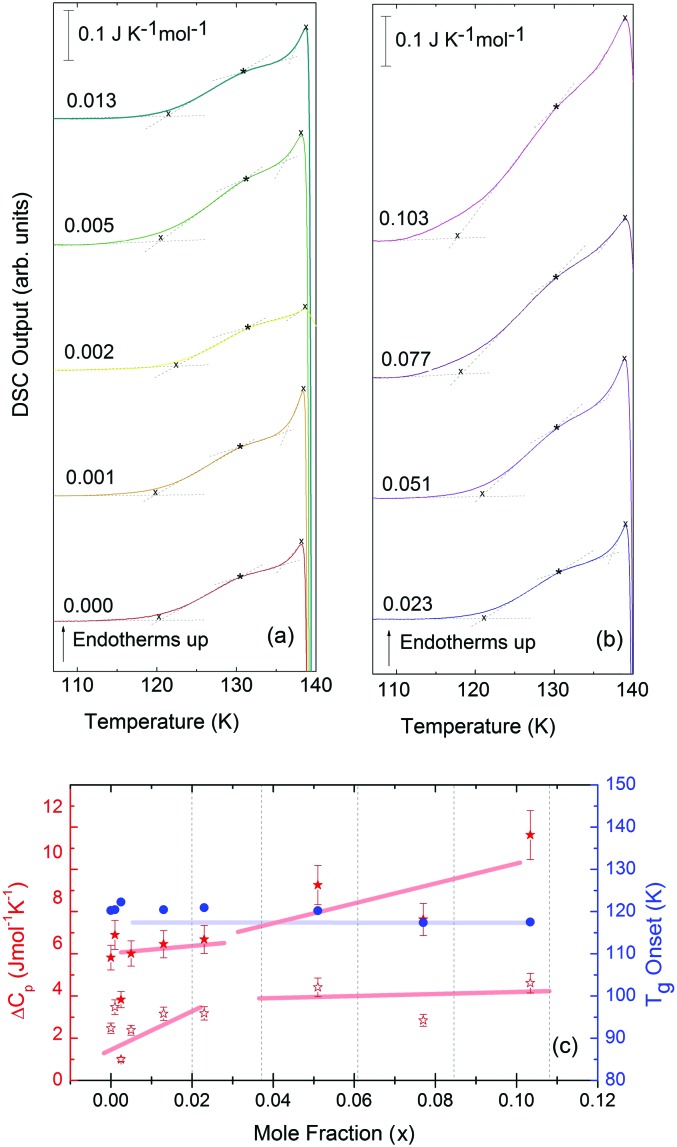
(a and b) DSC scans recorded at a rate of 30 K min^–1^ of the eHDA samples, concentrations ranging from *x* = 0 to 0.103. The glass transition can be seen within this temperature range, prior to the HDA to LDA transition. (c) Onset glass transition temperature (*T*_g_, blue circles), Δ*c*_p_ per moles of solution of full increase (Δ*c*_p_, full red stars) and Δ*c*_p_ per moles of solution of first step increase (Δ*c*_p_, empty red stars) as a function of LiCl concentration. Broad lines indicate trends as guides to the eye.

Turning now to the DSC scans of HDA's glass transition (after annealing at 1 bar and 108 K), we identify an endothermic event (see Fig. 7a and b) prior to the exothermic transition. This is assigned to the glass–liquid transition experienced by eHDA, similar to that reported by Amann-Winkel *et al.*^11^ in pure water, and reproduced here (see *x* = 0 scan). Because this endothermic effect is reproducible, it cannot be assigned to processes that involve restructuring of the HDA surface or annealing of microcracks, given that these processes would lead to an exothermic event due to a decrease of the system's free energy. In order to verify that HDA's glass transition is a bulk effect, we have performed these scans on finely powdered eHDA and on single chunks of the material, obtaining the same results as in ref. 12 and 13. Therefore, the HDA glass transition is clearly a bulk effect.

The heat capacity seems to increase in two steps – a flattening indicating the end of the first step (marked by tangents) followed by a spike is clearly evident in Fig. 7a and b. The origin of this phenomenon is unclear. Adiabatic cooling caused by the volume expansion of 25% may be one option to explain the spike. In this interpretation, the first step would be associated with the glass transition of HDA to HDL. In other words, at ≈132 K, the glass transition endpoint has been surpassed and equilibrated HDL has been accessed. It is then, however, unclear why the adiabatic cooling precedes the latent heat evolution accompanying the polyamorphic transition. The alternative interpretation is to assign both the first step and the spike to the glass transition in HDA. It is then unclear why the heat capacity increase flattens in the middle of the glass transition – it may be related to a decoupling of two types of motion that unfreeze in the whole glass transition range, *e.g.*, rotation and translation, but not only near the spike. The recent measurements by Perakis *et al.*,^47^ however, show clearly that diffusive motion is taking place in eHDA even below 132 K, supporting the interpretation of the spike as an adiabatic cooling event. We include values for both possible scenarios in our analysis in Fig. 7c. We here obtain an increase in heat capacity of 5.4 ± 0.5 J K^–1^ mol^–1^ (including the spike, 2.3 ± 0.5 J K^–1^ mol^–1^ without it) at *T*_g_ for the pure water sample, whereas Amann-Winkel *et al.* reported 4.8 J K^–1^ mol^–1^.^11^ This difference is presumably due to the different heating rate and to different treatment of the sample inside the DSC instrument and slightly different sample preparation beforehand. In Fig. 7c it can be seen that the glass transition temperature in eHDA is unaffected by the LiCl content, whereas the change of heat capacity at the glass transition is nearly constant up to *x* = 0.02, and it increases at higher salt contents. This suggests that the salt does not affect the phenomenon. The relatively large Δ*c*_p_ observed seems to indicate that the motion resulting in the endotherms reported in Fig. 7 cannot be due to an orientational glass transition, but indeed to a glass-to-liquid softening. This is explained since the associated thawing of an orientational glass transition caused by hydrogen atom mobility on an H-bond network fulfilling the Bernal–Fowler ice rules generates an increase in heat capacity of ∼1 J mol^–1^ K^–1^,^48^ and the effect observed here is about five times as large (including the spike).

#### 3.3.2 Water's first glass transition: LDA → LDL

**Fig. 8:**
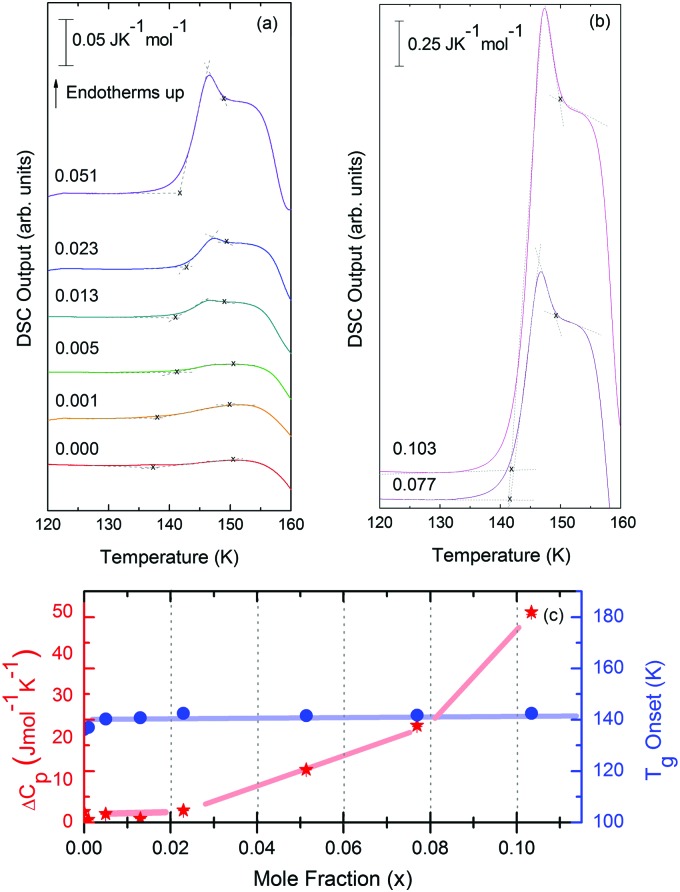
(a and b) DSC scans recorded at a rate of 30 K min^–1^ of LDA samples, concentrations ranging from *x* = 0 to 0.103. The glass transition can be seen within this temperature range, prior to the LDA to I_c_ transition. (c) Onset glass transition temperature (*T*_g_, blue circles) and Δ*c*_p_ per moles of solution (Δ*c*_p_, red stars) as a function of LiCl concentration. Broad lines indicate trends as guides to the eye.

Fig. 8a and b shows the isobaric heating scan of the LDA obtained by previously heating the eHDA beyond the polyamorphic transition to 145 K and recooling the resulting LDA sample to 93 K. The pure water sample exhibits a glass–liquid transition at 137 ± 2 K, in accordance with the literature.^11^,^28^

For concentrations higher than *x* = 0.005, this transition shifts to higher temperatures and is found near 140 K prior to crystallization. The measured *T*_g_ is almost unaffected by salt concentration, as shown in Fig. 8c. This is consistent with the behavior of salty-HDA samples but it differs from the behavior observed (see Fig. 1, ref. 44) for the hyperquenched LiCl aqueous solutions studied by Hofer *et al.*,^49^ where a minimum in *T*_g_ at *x* ≫ 0.04 followed by a sudden increase at a value close to 140 K is observed over the interval 0.05 < *x* < 0.15. The change in heat capacity increases much more with salt content, and it reaches about 70 J mol^–1^ K^–1^ at *x* = 0.103. Mayer *et al.* reported Δ*c*_p_ ≫ 20 J mol^–1^ K^–1^ for a hyperquenched solution with *x* = 0.083,^50^ in good agreement with the results of Fig. 8c. For comparison, at *x* = 0.103, the change in heat capacity amounts to only 6 J mol^–1^ K^–1^ in the case of the eHDA glass transition (Fig. 7c). With the exception of the pure water case where the glass transition is interrupted by the crystallization exotherm, all other glass transitions have a clear end point followed by a plateau. Assigning also this glass–transition to a softening and transition to the liquid, the state of the water in the plateau region is LDL. This plateau region is inaccessible in pure water samples, but now becomes accessible and stabilized by the presence of the ions at mole fractions *x* < 0.05. That is, dilute solutions of LiCl are required to reveal the endpoint without interference of the glass transition of the eutectic LiCl solution. This was not possible in earlier studies on vitrified solutions due to crystallization of dilute solutions.

### 3.4 Width of the glass transitions

**Fig. 9:**
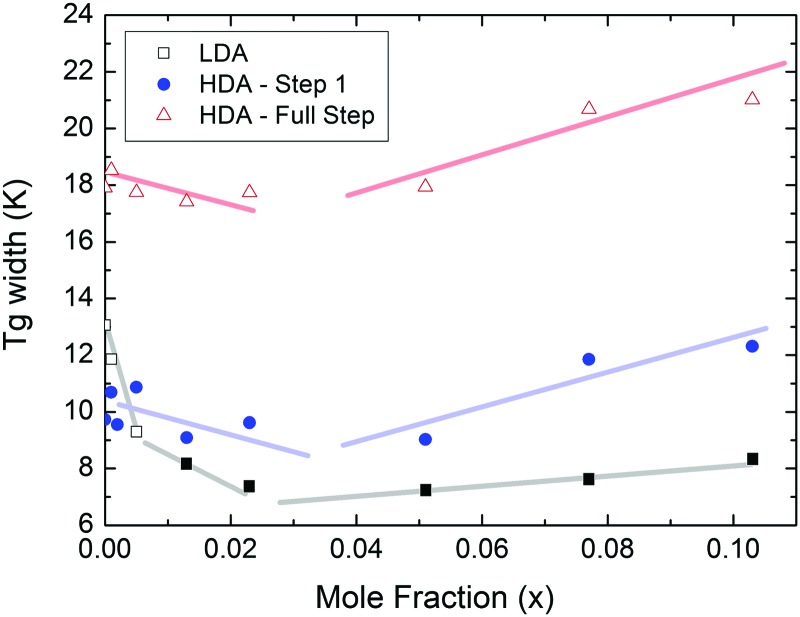
Full width of the full glass transition (triangles) and width of the first step (circles) in HDA as extracted from Fig. 7, and the full width of the glass transition in LDA (squares) samples as extracted from Fig. 8. Open symbols denote apparent widths (*i.e.*, end point not seen) and full symbols for real widths. Thick lines are guides to the eye.

At *x* = 0.005, the endpoint for LDA's glass transition moves into the window prior to crystallization, and it can be easily recognized at *x* = 0.103 in Fig. 8. For HDA, two endpoints can be defined, depending on which interpretation for the spike is favored. Fig. 9 summarizes the width of the glass transition of all samples studied here. For LDA, the apparent width is reported (open squares) for samples in which the endpoint cannot be accessed, and the full width is reported for samples in which the endpoint is seen (full squares). For HDA, the full width is shown assuming the plateau to indicate HDL (blue circles), whereas the apparent width is shown assuming the spike to be part of the glass transition rather than being adiabatic cooling (open triangles).

Since the onset temperature does not change due to the presence of the salt, its effect is to reduce the width of the glass transition. The full-width of the LDA glass transition is about 7.7 ± 0.5 K for all measurements at *x* > 0.01, so that the end-point of *T*_g_ can be resolved in the scan. However, the full width increases very rapidly at *x* < 0.01 to values higher than 12 K, so that only apparent widths can be determined from DSC scans, with the real width being even larger. In the case of HDA the first interpretation results in a full-width of about 10 K, and the second interpretation of 18 K for pure water and more dilute solutions. The relative width of the glass transition Δ*T*_g_/*T*_g_ amounts to 16% for the latter case. This is much larger than those observed for both fragile and strong liquids, and hence this is a strong argument against the spike being part of the glass transition. Therefore, we suggest the interpretation that the spike is due to adiabatic expansion and that only the first endothermic step is related to the unfreezing of translational diffusion in HDA. Using this interpretation, the relative width of the glass transition in HDA amounts to 9%, in good agreement with the relative width for the glass transition in LDA. This solves the question of the width of the glass transition in pure water and how much the heat capacity would increase if crystallization did not interfere. Upon extrapolating the width of the glass transition observed here to the pure water case, it becomes clear that crystallization interferes very close to the glass transition endpoint, somewhere in the region where the overshoot effect appears. That is, the apparent width of the glass transition of about 12 K and the apparent increase in heat capacity of about 1 J mol^–1^ K^–1^ found in earlier work^24^,^25^ represent very good values for the real width and real increase. Claims that the real values could be much higher can be refuted on the basis of Fig. 8. Furthermore, the glass transition width for HDL in the pure water limit is lower than for LDL. This suggests that HDL is a less strong liquid than LDL, which was shown to be one of the strongest liquids known (ref. 11). If the spike was part of the glass transition, then HDL would be even stronger than LDL, contradicting the earlier results.

## 4 Conclusions

In summary, we have observed direct evidence of a glass-to-liquid transition in LiCl HDA-type solutions. Earlier, Yoshimura and Kanno^31^ had inferred from Raman spectroscopic data that the glass possibly transforms to a high-density supercooled liquid (HDL) prior to transformation into the low density state. In this low-density state, we here observe a second glass transition that is distinct from the one in LiCl–HDA. The plateau region associated with the appearance of a supercooled liquid (likely to be LDL in LDA-type samples) clearly separates the glass transition domain from the crystallization exotherm. This provides strong evidence for the occurrence of two distinct liquids, where the addition of salt allows access to the (single metastable) low-density liquid (LDL) in a way that crystallization does not interfere prior to reaching the glass transition endpoint. Also, the (doubly metastable) high-density liquid (HDL) can be accessed before the polyamorphic transition interferes. It is now clear that for LDL, crystallization interferes just before the glass transition endpoint in pure water. Furthermore, HDL is a slightly less strong liquid than the superstrong LDL, as evidenced by the slightly narrower HDL glass transition width.

## Conflicts of interest

There are no conflicts to declare.
